# The influence of personal and environmental factors on professionalism in medical education

**DOI:** 10.1186/1472-6920-7-29

**Published:** 2007-08-30

**Authors:** Colin P West, Tait D Shanafelt

**Affiliations:** 1 Division of General Internal Medicine, Department of Medicine, Mayo Clinic College of Medicine, Rochester, MN, USA; 2 Division of Hematology, Department of Medicine, Mayo Clinic College of Medicine, Rochester, MN, USA

## Abstract

**Background:**

Professionalism is a critical quality for physicians to possess. Physician professionalism has received increased attention in recent years, with many authorities suggesting that professionalism is in decline. An understanding of the factors contributing to professionalism may allow the development of more effective approaches to promoting this quality in medical education.

**Discussion:**

We propose a model of personal and environmental factors that contribute to physician professionalism. Personal factors include distress/well-being, individual characteristics, and interpersonal qualities. Environmental factors include institutional culture, formal and informal curricula, and practice characteristics. Promotion of professionalism requires efforts directed at each of these elements.

**Summary:**

One responsibility of medical education is to foster the development of professionalism among its learners. Both personal and environmental factors play a role in physician professionalism. Accordingly, institutions should consider these factors as efforts to promote physician professionalism evolve.

## Background

"One of the mysteries of illness is that no one can be healed by anyone whose emptiness is greater than their own."

Mark Nepo [1]

Professionalism is a critical quality for physicians to possess. Professionalism requires integrity, honesty, compassion, a commitment to keeping current with medical advances, the ability to communicate effectively with patients, and respect for patient autonomy. Physician professionalism has received increased attention in recent years, as changes in health care delivery, technological advances, and an ever-increasing level of biomedical complexity have altered the way physicians care for patients [2-7]. Here, we review the elements of professionalism and the role of professionalism in medical education. We discuss personal and environmental factors influencing the professionalism of individual physicians and what is known about how to promote professionalism. We focus particular attention on factors impacting the humanistic underpinnings of professionalism.

### Professionalism: defining the elements

The term 'professionalism' is derived from the Latin *professus*, meaning to have declared publicly [8]. Originally relating to an act of openly declaring or publicly claiming a religious belief or faith, this term has come to represent adherence to the values professed by individuals engaged in the practice of a specific discipline such as religion, law, or medicine. Despite the well-recognized importance of professionalism in the field of medicine, a precise definition of the values that comprise physician professionalism has proved elusive [9-13]. One comprehensive attempt to define physician professionalism is the Charter on Medical Professionalism, developed through a joint effort of the American Board of Internal Medicine, the European Federation of Internal Medicine, and the American College of Physicians [14]. This work identifies the primacy of patient welfare, patient autonomy, and social justice as the three core principles of professionalism in the field of medicine and proposes ten responsibilities individual physicians must uphold to embody this quality.

Other authors have more explicitly cited humanistic values including respect for others and empathy as additional elements of professionalism in medicine [4,7,9,10,13,15-23]. The term "empathy" refers to the ability to understand patients' perspectives of their experience and to effectively communicate this sense of understanding to provide support. These attributes have been shown to correlate with improved patient care and have been termed "the foundation of the patient-physician relationship" [[24], pg. 867]. Patient perceptions of physician empathy are intimately related to their overall satisfaction with their medical care and their ratings of physician professionalism [25-28]. A lack of empathy among physicians has also been found to predict poor clinical performance [29-31]. Thus, interpersonal aspects of professionalism are integral to professionalism as a whole.

### The role of professionalism in medical education

Several organizations involved in the accreditation of physicians and training programs have labored to promote physician professionalism. In 1995, the American Board of Internal Medicine published its Project Professionalism, an effort to define professionalism, raise awareness of its importance, and develop strategies for fostering and evaluating physician professionalism [17]. One year later, the American Association of Medical Colleges identified professionalism as a fundamental quality of physicians in its Medical School Objectives Project, a consensus statement formalizing the attributes required of all graduating U.S. medical students [18,32]. The Accreditation Council for Graduate Medical Education has also cited professionalism as one of six fundamental competencies all physicians in training must develop, and has mandated that residency programs both train residents to be professional and measure resident development of this quality [19]. Other professional organizations have made similar proposals [33,34].

These widespread efforts emphasize the recognized importance of professionalism for physicians. This is underscored by research showing that professionalism impacts the quality of the medical care physicians provide as well as patients' satisfaction with their care [25]. To most effectively promote professionalism, an appreciation for the personal and professional factors that influence professional development is necessary.

## Discussion

### Personal factors affecting professionalism

**Figure 1:**
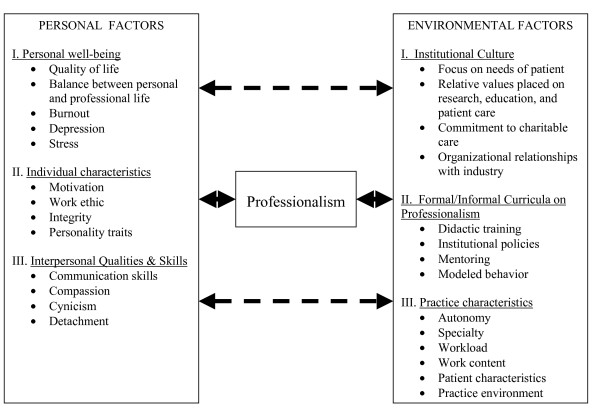
Model of personal and environmental factors contributing to physician professionalism.

Humanistic qualities, integrity, and strong work ethic are elements of the selection criteria for acceptance into medical school, and one would expect that students begin their medical training with great capacity for professionalism. Unfortunately, studies suggest that crucial elements of professionalism, including empathy and humanism, decline rather than develop during the medical school and residency training process [53-55]. As described below, this erosion of professionalism appears to be related in part to personal factors, including personal distress experienced during training, individual characteristics and personality traits, and interpersonal skills (Figure 1).

Although some degree of stress is a natural part of physician training and may promote a physician's ability to perform under pressure, it is increasingly recognized that many medical trainees and clinicians experience unhealthy levels of distress [56,57]. It has been suggested that the fundamental element of professionalism is that the interests of patients and society supercede physician self-interest [9], but physician distress is associated with a decrease in compassion and empathy [49,54,58-60]. This supports the intuitive concept that it is difficult for physicians to put the needs of others first while experiencing personal crisis.

In addition to the life stressors experienced by all individuals in society, physicians face a unique combination of profession-specific stressors including financial burdens related to tremendous educational debt, sleep deprivation, dealing with patient suffering and death, unrealistic patient expectations, and work-life imbalances related to limited control over their schedules (particularly during residency and fellowship training) [2,17,50,54]. Personality traits and learning style further influence how these factors affect individual physicians [63]. The stress caused by these factors can contribute to physician burnout, a syndrome of emotional exhaustion, depersonalization, and a sense of low personal accomplishment that leads to decreased effectiveness at work [49,50,64-67]. Numerous studies indicate a link between burnout and erosion of physician professionalism [49,50,68,69], and imply that burnout contributes to suboptimal patient care practices [31,62]. Unfortunately, these studies also demonstrate that burnout is common in physicians at all levels of training and practice, from medical students to department chairs [70,71]. Particularly high levels of burnout are observed among resident physicians [31,62], which may contribute to the "culture of cynicism" observed in supervising residents by medical students and interns.

This growing body of evidence suggests that the concept that professionalism inherently requires placing patient needs before those of the physician is valid only within a limited context, and that carrying this principle to the extreme (i.e., to the point that the physician becomes personally depleted) actually undermines professionalism. Consistent with this premise, a small but increasing literature suggests that promoting enhanced well-being in physicians may enhance their ability to provide compassionate care to patients [50,58,60,72]. For example, the patients of physicians with high job satisfaction are more satisfied with their medical care, perhaps relating to better communication and/or more empathy [58]. Research directly measuring physician well-being and empathy with validated survey tools has also implied that increased well-being may enhance and promote empathy [60].

Physicians, however, often fail to appreciate the relationship between their personal well-being and the care they provide. For example, when asked to list attributes associated with professionalism, residents in one study rated "balance between personal and professional life" last among 28 reported characteristics [73], despite numerous reports demonstrating that such balance is essential to prevent burnout and its negative effects on patient care, empathy, and professionalism [66,67].

Recognition that physician distress negatively impacts both physician and patient has led to several recent reforms in the physician training process, including regulation of trainee duty hours [74-76]. These reforms have sparked spirited debate regarding their potential effects on resident professionalism [16,77-83]. Many authors contend that spending fewer consecutive and total hours with patients will lead to a decline in professionalism. Others argue that the historical systems of training are conceptually flawed, and recommend a reassessment of how we balance patient care, education, and personal well-being during physician training. Although some have suggested that these differences of opinion represent a generational divide [16], at the heart of the issue are attitudes concerning the role of physicians' personal health and well-being in professional development and quality of patient care.

A process model that further defines the relationships among empathy, well-being, patient care, and professionalism has been described by Larson and Yao [84]. In this model, empathy is likened to emotional labor, requiring both internal and external emotion management. Deliberate modulation of external emotions by physicians, or so-called "surface acting", may improve patient perceptions of empathy and professionalism. The guiding of physician behavior by internal emotions, or "deep acting", may allow the development of more substantial connections and true "empathic engagement" with patients [85]. Ultimately, the energy physicians can devote to emotional labor is a finite resource. Personal renewal through mindful practice and nurturing of personal interests and relationships can minimize the risk of internalizing patient distress that is inherent in establishing close emotional ties with patients [86]. The interactions among these factors illustrate the importance of attention to the personal needs of both patient and physician to maximize professionalism.

### Environmental factors affecting professionalism

In addition to personal factors, a number of organizational, environmental, and societal factors shape the culture of the medical profession and profoundly influence the professionalism of individual physicians [5,87]. These factors include institutional customs, the formal and informal curricula, and characteristics of the practice environment such as workload, specialty, practice setting, patient characteristics, and malpractice concerns [61,62] (Figure 1).

Institutional efforts to foster professionalism include mentoring programs, formal training in biomedical ethics, and institutionally supported volunteer experiences. Unfortunately, despite the many virtues of the current system of medical education a "gradual disintegration of the education community" [[4], pg. 610] has been recognized, and may result in a shortage of effective role models for physicians in training and modeling of undesirable behaviors that greatly impact the professionalism of trainees and faculty [88].

The perceived erosion of institutional commitment to professionalism among academic medical centers has in part been attributed to a number of financial and regulatory challenges that threaten traditional academic values. Historically, academic centers have relied upon reimbursement rates commensurate with the high quality, highly specialized services they provide. Increasingly, however, teaching institutions find it difficult to secure the compensation necessary to maintain their academic missions. Because patient care costs at academic centers are typically higher than at other centers as a result of greater patient complexity and increased demands for charitable care, these centers are at particular risk [89-92]. Additionally, while trainee duty hours have been reduced, few academic centers have the financial means to hire additional providers to complete the work previously performed by residents and fellows. At many centers, this has resulted in increased clinical demands on academic faculty, threatening the time they can devote to medical education and academic research and undermining their ability to serve as role models for physicians in training.

The financial pressures created by decreased government funding of research and education, increasing patient care costs, and decreased reimbursement for medical care have also led many academic medical centers to pursue greater collaborations with industry [89,93]. While academic medical centers and industry share a common goal of advancing the science of medicine, their motives for this pursuit can at times differ markedly (i.e., science to achieve profit versus science to enhance the public good). This can lead to potentially significant conflicts of interest for academic institutions and their faculty, shifting priorities away from education and patient-centered care. Most institutions take great care to foster an environment of professionalism for their faculty by developing specific policies governing such relationships to minimize conflicts of interest [94]. Despite these efforts, numerous studies have demonstrated that relationships between academia and industry may result in adverse events such as data-withholding and selective publication of results [89,94-99]. Such occurrences represent a violation of the public trust and are inconsistent with professionalism. These relationships illustrate the impact organizational factors may have on professionalism.

Direct marketing to faculty and physicians in training by the pharmaceutical industry represents another environmental threat to professionalism. While gifts from industry to individual physicians or academic centers are often portrayed as inconsequential promotional activities, they establish an implicit relationship between medicine and industry with associated *quid pro quo *obligations [100]. Clinicians typically deny that such marketing and gifts influence their prescribing behaviors, but there is substantial evidence to the contrary [100-102]. Despite the fact that they accept these gifts, many physicians believe that pharmaceutical representatives prioritize product promotion above patient welfare and may even exercise unethical practices to promote their products [101]. Nevertheless, academic medical centers routinely allow pharmaceutical marketing directly to medical students and residents. Nearly all medical students report being asked or required by supervising faculty to attend industry-sponsored functions [102], suggesting that institutional policies regarding conflicts of interest are often not well integrated into clinician behaviors. Such competing influences on professional development can be confusing for trainees, who are faced daily with mixed messages regarding professional values.

In fact, trainees report that the values exhibited by their teachers and institutions directly impact their own professionalism [35,39]. Unfortunately, a significant percentage of physicians and medical students are dissatisfied with their formal training in professionalism and report experiencing a culture of cynicism during training [7,103-107]. A majority of students and residents report witnessing peers and supervising physicians refer to patients and colleagues in a derogatory manner, and also report being personally mistreated by peers, educators, and patients [4,108-111]. Perhaps related to these experiences, multiple studies have found that humanistic attitudes, particularly empathy, decline throughout medical school and residency training and that levels of anger and depression increase [53-55,110,112].

These observations emphasize the important roles of the "formal", "informal", and "hidden" curricula in how physicians develop and maintain professionalism [7,105,106]. Hafferty has defined the formal curriculum as "the stated, intended, and formally offered and endorsed curriculum", the informal curriculum as "an unscripted, predominantly ad hoc, and highly interpersonal form of teaching and learning that takes place among and between faculty and students", and the hidden curriculum as "a set of influences that function at the level of organizational structure and culture" [[106], pg. 404]. The informal and hidden curricula teach professionalism through modeled behaviors and organizational cultures which, unfortunately, often stand in stark contrast to the conduct promoted in formal professionalism coursework. The influence of these curricula on professionalism in medicine is insidious but powerful. The disconnect between the formal and informal/hidden curricula further confuses current and future clinicians and can promote cynicism, since students and physicians learn that the ideals espoused during training do not match the realities seen in daily practice among their peers and role models [7]. Academic medical centers and training programs must respond to these issues if they are to effectively revive a culture of professionalism for physicians in training [6,113-115].

### Promoting professionalism

While nearly all U.S. medical schools now have some form of professionalism curriculum, how best to promote and evaluate professionalism is unclear [35,36]. A number of methods to foster professionalism in students and residents have been proposed, including formal coursework in ethics and humanism, development of faculty role models, mentoring of both students and faculty, requiring trainees to participate in the delivery of charitable care and community service, focused precepting in humanism, and providing opportunities for personal and shared reflection among physicians in training [4,20,33,34,37-42]. These efforts largely reflect the belief that professionalism is a characteristic that cannot be instilled effectively without the direct participation of the learner. Reports on the application of these techniques have been primarily descriptive in nature. Although they do not provide definitive evidence of effectiveness, the face validity of these approaches is sufficient to argue for their continued application while research aimed at delineating the most useful methods continues.

It should also be noted that efforts to measure the impact of these approaches have been limited by the fact that the best way to evaluate professionalism among physicians is unknown. A number of methods to measure professionalism have been described, including self-rating, peer and supervisor review, patient assessment, standardized patient encounters (e.g., Objective Structured Clinical Examinations and Standardized Patients), written examinations, response to clinical vignettes, survey tools, and portfolio development [4,10,20,33,38,43-46,116,117]. Other unique proposals include the use of "humanism connoisseurs" to provide specialized evaluation of professionalism [47].

The appropriateness of each of the above approaches to teach and assess professionalism likely varies by clinical setting, and has been the subject of several recent reviews [10,44,45,48]. To date, however, few strategies for teaching or assessment of professionalism satisfy basic criteria for content validity, reliability, and feasibility [48], and this remains an active area of medical education research.

Many recommendations for restoring and maintaining the ideals of medical professionalism have been made [4,6,7,13,15,20,33,40,61,114,118]. These have focused in large part on the importance of effective role models, and have emphasized integrated, interdisciplinary curricula for professionalism education rather than purely lecture-based approaches. Organizational reforms to support professionalism both in word and deed are necessary, including efforts to promote a culture of caring rather than informal and hidden curricula that model cynicism. Efforts to promote and support the personal well-being of physicians are crucial [50], and could be enhanced by emphasizing our responsibility to care not just for our patients but also for ourselves and for our colleagues.

For example, prioritizing values in support of relationships, self-care, and rational work limits is likely to promote physician well-being [49,119-121]. Approaches that foster self-awareness and reflection, including Balint groups, may also be helpful [85,119,122,123]. Organizational efforts should be directed at supporting these goals, promoting work-life balance, and maximizing workplace autonomy [50].

Efforts to promote professionalism must also consider the impact of the practice environment on the professionalism of individual physicians. Organizational policies guide physicians as they navigate the many challenges to professionalism that are part of modern medical practice. Medical institutions must remain committed to societal needs including charitable care, just allocation of resources, and avoidance of conflicts of interest. Government funding for research, education, and patient care must ensure that the commitment of medical centers to core professional values does not compromise financial viability and necessitate relationships that conflict with these values.

Institutional principles consistent with the ideals of professionalism serve as visible rebuttals to the informal and hidden curricula from which so many students and physicians learn their professional behavior. Such principles should not be mere slogans but must accurately reflect the culture of care institutions seek to promote. Medical leadership has a particular duty to protect junior learners from negative influences during the formative period of their professional development [124,125]. Academic medical centers must make an honest appraisal of what messages are being conveyed to medical students and residents via the hidden curriculum at their institution. Where necessary, re-training of faculty and senior residents may help redirect a culture of cynicism toward a humanistic environment of mutual support in which compassion and professionalism may thrive.

We believe the concept that caring for the patient and society above caring for oneself is sustainable only within the context of appropriate limits and attention to physicians' personal needs. Physicians are themselves therapeutic instruments that require maintenance and renewal to remain effective [122,126,127]. Substantial evidence now demonstrates that a physician's ability to empathically care for patients in fact depends in large part on the physician's ability to care for himself or herself [49,60,62]. As such, the compassion and emotional investment physicians have to offer their patients are limited resources that must be replenished. Without such renewal, continued personal sacrifice is likely to lead to emotional depletion, depersonalization, and cynicism. As discussed above, efforts to promote professionalism must take into account the importance of both personal and organizational/environmental influences, and must be crafted with an understanding of the relationships among these influences.

## Summary

Medical professionalism has come under increasing scrutiny in recent years, with many authorities suggesting that the professional attributes of physicians are in decline. Multiple factors, both personal and organizational, influence the professionalism of individual physicians.

As a profession, we must improve recognition of the relationship of physician self-care to our ability to care for patients. This should not be seen as conflicting with the primacy of patient welfare, but rather as a necessary process to preserve and enhance our ability to meet the needs of our patients. Several authors have called for an increasing commitment to self-awareness and personal health, and we join them in rejecting a false concept of professionalism that results in personal distress [6,16,39,51,128].

Despite a mandate by the Joint Commission on Accreditation of Healthcare Organizations that hospitals have processes to promote physician wellness [129], the literature provides little guidance as to how to accomplish this effectively [50,122,130]. Additional research in this area is needed if we are to recapture and enhance physician professionalism. Further research into physician wellness promotion strategies is required, and outcome studies are necessary to evaluate the impact of these strategies on both personal well-being and elements of physician professionalism.

Environmental influences also play an integral role in professionalism. Modeling of and support for desirable behaviors in the informal and hidden curricula should support the formal curriculum for professionalism. Improving the concordance among these curricula would be expected to result in a work environment more conducive to the professional attributes society and we ourselves expect of physicians.

The goal of the medical professional is the care of patients. To do so in a compassionate and empathic manner requires that physicians themselves be well. Accordingly, decisions at both the individual and institutional level made without consideration for both the patient and the physician ultimately serve neither. In the end, the best interests of the patient and the physician cannot be separated if we wish physicians to be effective healers.

## Competing interests

The author(s) declare that they have no competing interests.

## Authors' contributions

CW contributed to the conception of this paper and wrote the manuscript. TS contributed to the conception of this paper and participated in detailed revision of the manuscript. Both CW and TS read and approved the final manuscript.

## Pre-publication history

The pre-publication history for this paper can be accessed here:


